# A Hierarchical Taxonomy of Test Validity for More Flexibility of Validation

**DOI:** 10.3389/fpsyg.2018.00972

**Published:** 2018-06-15

**Authors:** Jinsong Chen

**Affiliations:** Department of Psychology, Sun Yat-sen University, Guangzhou, China

**Keywords:** hierarchical taxonomy, test validity, construct domain, context specific, score use

## Abstract

Test validity lies at the core of educational and psychological testing, but there are controversies about what test validity is and how test validation should proceed. This paper develops a taxonomy to redefine test validity with hierarchical levels. On the basis of testing foundation, the hierarchy includes operational, measurable, realizable, and useful levels, which result in testing consequence. With the help of a context-specific construct, different levels of test validity, and different types of score use, the proposed taxonomy offers more flexibility for test validation. It can also shed light on the interpretations of important testing concepts and help streamline test development. Real-life examples are given to demonstrate the usefulness of the taxonomy across different settings.

## Introduction

It is generally agreed that validity is the most fundamental consideration in educational and psychological testing (American Educational Research Association et al., 1999, 2014; Kane, 2006). However, validity is also one of the most controversial issues in testing and assessment, with disagreement about what test validity is and how test validation should proceed (e.g., Kane, 2001; Borsboom et al., 2004; Lissitz and Samuelsen, 2007; Hood, 2009; Lissitz, 2009; Cizek, 2012). Since validity is vital to the testing discipline, the influence of the issue is far-reaching in testing and psychometrics. For instance, the controversies give rise to disagreements and confusions around the key concept of psychological construct (e.g., Michell, 2013; Slaney and Racine, 2013; Slaney and Garcia, 2015). In general, it is still true that “[w]hile there are some authors who feel that validity is a somewhat settled concept others disagree with that position” and “validity is still in great need of intellectual advancement” (Lissitz, 2009, p. 3). Challenged by the issue, this paper attempts to develop a hierarchical taxonomy of test validity based on the recently advanced concept of context-specific construct (Chen, 2017). Before proceeding, it is necessary to introduce some background that can give rise to the context-specific construct and the proposed taxonomy. However, the brief introduction is not intended to be a comprehensive review of the validity controversies, and interested readers should refer to the above references for that purpose. Additional information or viewpoints regarding the validity issue can be also found elsewhere (e.g., Sartori and Pasini, 2007; Borsboom et al., 2009; Michell, 2009; Pellegrino et al., 2016).

Many disagreements stem from two different theoretical approaches (Hood, 2009): ontological vs. epistemological, which correspond to the relatively divergent and mainstream classifications of validity conceptualization, respectively, to some extend (Lissitz, 2009). In the traditional or ontological approach, validity is the property of the test or test score; that is, a test is valid if it measures what it purports to measure (Kelley, 1927; Cattell, 1946). According to Borsboom et al. (2004), stating that the test score is a valid measure of the psychological attribute implies both the theoretical existence of the attribute and its causation of the test score. Psychological measurement is a natural extension of physical measurement, with psychological attributes understood as analogous to physical attributes. This approach to test validity is elegant and concise but increasingly unpopular in psychological and educational testing for three reasons. First, it requires strong theoretical or empirical support for the existence of the attribute and its causation of the behavioral responses—support that is increasingly challenging in social and behavioral fields. Second, although some psychological attributes can be largely independent of context, many others are intertwined with contextual factors, and the related score meaning may not be independently defined or validated. Third, since test validity is not different from research validity in general, there is no universal way to guide the validation process and little useful information to inform test development.

In the contemporary or epistemological approach, validity refers to the degree to which empirical or theoretical evidence supports the interpretation and use of the test score (Messick, 1989; American Educational Research Association et al., 1999, 2014; Kane, 2006). The ontological question of “what is validity” is mixed with the methodological question of “how to validate” (Borsboom et al., 2004; Cizek, 2012). Moreover, validity or validation is considered unitary rather than fragmented under the concept of construct validity (Loevinger, 1957), whereby different types of evidence and social consequences are summarized and judged in an integrative way (Messick, 1989). A construct can be considered shorthand for regularities or patterns of behavior, and score interpretation in terms of the construct can be circular (Kane, 2006). Construct or score interpretations are inseparable from the testing purpose and the circumstances of the observable behavior. Although pragmatic and useful in informing test development, this approach also suffers from certain concerns. First, due to the lack of causal explanation of the test score, the approach runs a risk of slipping into the so-called weak program where any evidence connected to the test score is considered relevant to validity, making validation purely empirical and score interpretation exploratory. Second, without a clear ontological concept and theoretical rationale, the approach is open ended, and it is not clear how much evidence one should accumulate to determine the degree of validity. Third, the different types of evidence are qualitatively different and can be incompatible under the unitary concept of construct validity (Cizek, 2012). Value judgment can be easily confounded with analytical evidence when evaluating the consequences of testing.

Although the two approaches appear to be dialectically opposed, they can complement each other (Hood, 2009). In recent conceptualizations of validity, there have been increasing attempts to draw ideas from both approaches (e.g., Lissitz and Samuelsen, 2007; Cizek, 2012). Viewed from the nature of the construct or attribute,^1^ the two approaches represent two ends of the context-dependency continuum in the domain of observation, ranging from context-independent to context-inseparable. On the independent end, the construct exists without reference to any contextual factor theoretically. This independence implies that valid measures of the construct will be reliable and meaningful regardless of the circumstances. Accordingly, score meaning based on the theoretically grounded construct can be independently defined and validated in any circumstance, and hence validity is a property of the test score. On the inseparable end, the construct can be considered shorthand for patterns or regularities of observable behavior in the domain of observation and is inseparable from contextual factors. Construct or score meaning is partially shaped by the intended use of the test score. This view implies that score interpretation is inseparable from the circumstances of intended use, and valid measures of the construct need to take into account the testing purpose or score use. Accordingly, validity is a property of score inferences based on the intended interpretation and use, resulting in a unitary approach to validation.

The two approaches can be bridged by assuming a context-independent construct within a specific boundary. With the help of a context-specific construct (Chen, 2017), this article presents a hierarchical taxonomy to redefine test validity. With its different levels of test validity and different types of score use, the proposed taxonomy can offer more flexibility in test validation. The approach also allows for the reconsideration of relationships among key testing concepts—testing purpose, construct, and instrument—and how they can be aligned with each other during test development. This article begins by elucidating and extending the concept of a context-specific construct. It then scrutinizes the structure of the hierarchical taxonomy and explains how important testing concepts can be reinterpreted within the taxonomy. Afterwards, the article illustrates the usefulness of the taxonomy across different settings using real-life examples. The article concludes with a discussion of issues related to applying the proposed taxonomy.

## The Context-Specific Construct

This section expounds and extends the concept of context-specific construct (Chen, 2017). A construct is context specific when it can be uniquely defined within the boundary of a specific context. Most if not all context-specific constructs can be measured by one test score^2^. Namely, valid measures of the construct will be reliable and meaningful within the context. With the help of the testing foundation, which can be theoretically grounded and/or practically driven, the unique construct domain and corresponding score meaning can be shaped by taking into account contextual factors such as age and culture. The theoretical foundation comes from theory or research findings, which explain the causality of the test score. In contrast, a practical foundation supports the need for the test score based on practical (e.g., legal, professional, educational, or clinical) regulations or requirements. The shaping of the construct domain can be theoretically dominated given a strong theoretical foundation (e.g., executive functions, intelligence), or otherwise can be practically driven (e.g., accounting proficiency, math achievement). In any case, the legitimacy of the testing foundation is prerequisite to valid measure of the construct domain. Accordingly, one can argue that test validity is conditional.

The concept of a context-specific construct provides a way to bridge the two approaches at both ends of the context-dependency continuum. From a pragmatic or measurement point of view, this means that a construct is measurable only when it can be uniquely defined or operationalized within a specific context. Note that although the unobservable construct plays an important role in testing, the building block of social and behavioral science is the possible behavior within a specific context. On the inseparable end of the continuum, the construct domain can be highly contextualized due to the practical inclination of the testing foundation. For instance, when a certain degree of accounting proficiency is necessary for qualified practice based on accounting regulations in specific regions, the construct of accounting proficiency is criterion-referenced and should be defined with contextual factors such as region and language. In contrast, a testing foundation with strong theoretical support can be largely context independent, and the construct domain will be free from contextual factors.

Based on the testing foundation, a context-specific construct can be uniquely defined with three elements: population, structure, and content. The population element defines the target population with demographic or contextual features such as age, culture, place, and language that the test score is intended to be generalized to. The structure element defines the dimension and scale level of the construct (i.e., continuous vs. categorical), which can inform the structure and frame of reference (e.g., norm- or criterion-referenced) of the test score. In the case of a continuous construct (i.e., norm-referenced score), the normative group is implicitly defined by the population. In the case of a categorical construct (i.e., criterion-referenced score), categorical or criterion levels are external to the population and need to be qualitatively defined prior to testing, usually by subject experts. For instance, the construct of unidimensional math ability can be referenced to external criteria defined as mastery, partial mastery, and non-mastery. When the construct is multidimensional and criterion-referenced, the criterion levels for each dimension need to be defined individually. The content element endows the construct with substantive content (e.g., operational descriptions, behavioral patterns, or regularities). For a multidimensional construct, each dimension should be substantively defined. In large-scale assessments, the content element usually covers a wide range of behavior patterns and can be further structured or partitioned into a finer-grained size. For small tests, the content can comprise operational descriptions of the latent traits in simpler form. With these three elements, the construct domain and corresponding score meaning are uniquely defined. This framework also suggests the differentiation of score meaning and score use, as every test score should be defined once (i.e., unique construct domain) but can be used in multiple ways (e.g., to make predictions or decisions, to diagnose).

## The Hierarchical Taxonomy of Test Validity

**FIGURE 1:**
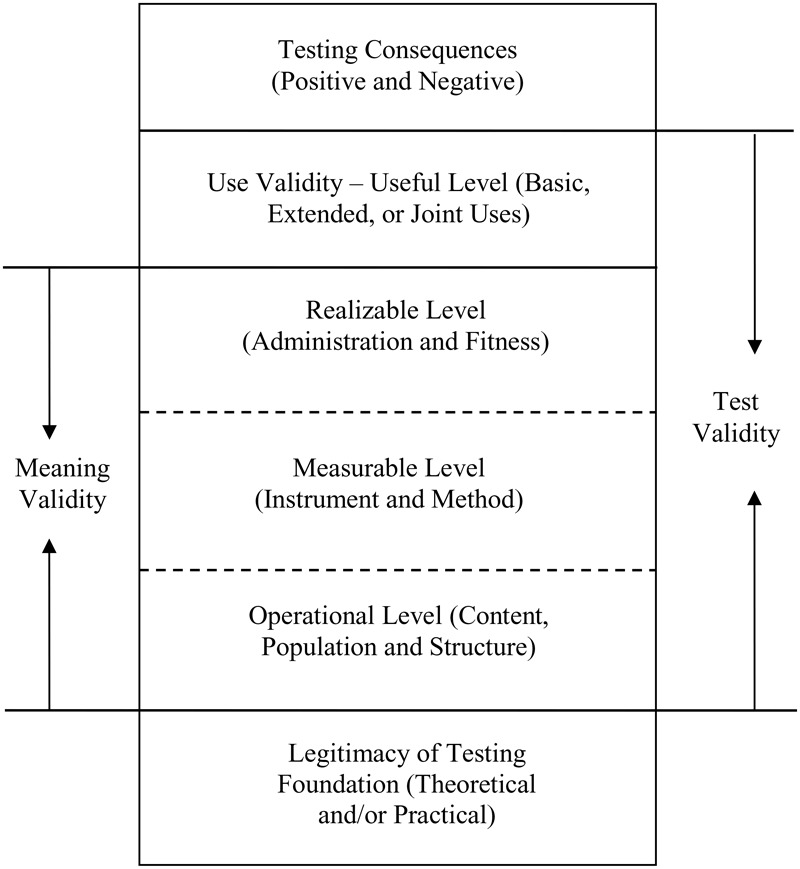
The hierarchical taxonomy of test validity.

Based on the concept of a context-specific construct, a hierarchical taxonomy of test validity is proposed, as shown in **Figure 1**. Test validity includes the validity of score meaning (i.e., meaning validity) and the validity of score use (i.e., use validity). Moreover, these two forms of validity are conditional on the legitimacy of the testing foundation and serve as a basis for different consequences of test use (i.e., testing consequences). The hierarchy informs the logical order of test validation and evaluation. Note that test validity is essentially scientific, whereas the testing consequences, including fairness, can be value-laden.

### Three Levels of Meaning Validity

The meaning validity consists of three hierarchical levels, which can be further partitioned into multiple claims that can be individually attested if needed. The operational level addresses the validity of the operational definition of the construct domain with the three elements (i.e., population, structure, and content). It aims to validate if the testing foundation is appropriately transformed into the operational definition.

The measurable level addresses the validity of the instrument (e.g., item, format, and specification) and the method used (e.g., model) to analyze the response data and derive the test score based on the operational definition. Valid instruments should transform the operational definition into appropriate items, response formats, and specifications for measurement. These can be any devices or procedures that are used to systematically sample examinees’ behavior with a standardized process (American Educational Research Association et al., 1999, 2014). Specifically, the items and response formats should be content representative, with irrelevant content as little as possible. The specifications include the testing conditions, standardized process, and examinee characteristics, all of which should be consistent with the operational definition, and especially the population element. When there are large discrepancies between the specifications and definition, additional evidence is necessary to justify the generalization. Valid methods should establish appropriate correspondence between the structure of the construct and the item responses. The methods can rely on any models based on classical test theory, generalizability theory, or item response theory.

The realizable level addresses the validity of the administration during testing and the fitness between the response data and the models. Valid administration means that the testing is appropriately administered to obtain the test score. It includes following the specifications to obtain the response data and the methods to score the responses. The model-data fitness serves as the final and integrative evaluation of meaning validity. In hierarchical order, the three levels address whether the construct is operational, measurable, and realizable with a specific test score.

### Useful Levels or Use Validity

Meaning validity is prerequisite to use validity, as a test score has to be meaningful before it can be useful. If the score meaning is valid, the test score can be used in different ways or for different purposes. When score use depends solely on score meaning, no additional variable or factor is involved, and this is considered basic use; the use validity is essentially the meaning validity. The involvement of any external variable or factor constitutes an extended use, requiring additional effort to validate the score use. When the test score is used to make decisions, for instance, one can distinguish between criteria-driven and selection-driven cases. Criteria-driven cases rely on the cutoff based solely on score meaning and can be regarded as basic use. Real-life examples are test-based professional or educational certificates. In selection-driven cases, the cutoff is at least partially driven by external variables (e.g., gender, race) or factors such as the number or proportion of examinees that one can admit and should be considered an extended use. Real-life examples are test-based employment or educational admissions. Note that since the score meaning is unique, so does its basic use. In contrast, there can be multiple ways of extended use, each of which should be validated individually.

Using multiple test scores together, each with its own construct domain, constitutes a joint use. In this case, the meaning of each test score is validated individually, whereas their joint use is validated jointly. This provides an alternative approach of test validation: Instead of validating different substantive contents with one construct domain (i.e., one test score), the contents can be divided first into multiple domains for validation of score meaning and then validated together for joint use. It is possible that the meaning of each test score is valid whereas their joint use is not. Joint validation can be especially helpful when the testing foundation is not homogenous across different substantive contents, which is not uncommon in large-scale assessments with an ambitious purpose and wide scope. In language assessments, for instance, the language construct with one test score often consists of different skills (e.g., reading, writing, listening, and hearing), the use of which is validated in a unitary way under the contemporary approach (Chapelle et al., 2010). Under the hierarchical taxonomy, the score meaning of each skill can be validated before validating the joint use of multiple test scores. Since the theoretical and practical foundations of the skills differ substantially, the proposed approach would be more reasonable. Note that the population element from different construct domains should at least overlap to some extent for a joint use of multiple test scores.

### Reinterpretation of Key Testing Concepts

#### Degree of Validity, Evidence, and Claim

Under the hierarchical taxonomy, the validation processes and related evidence are structured, which can be used either for the confirmatory or disconfirmatory perspective of test validation. Moreover, the hierarchy is ordinal and can be connected to the degree of test validity, since the validity of upper levels is conditional on that of lower levels, but the reverse is not true. For instance, an inappropriate instrument or method makes the test score meaningless, but would not affect the validity of the construct domain. Similarly, the construct is still measurable if invalid evidence is found only at the realizable level (e.g., inappropriate administration). Further, validation of lower levels should be prioritized over that of upper levels. Validity is always evidence-based, and the taxonomy can be divided into different claims or arguments, which can be supported or rejected with different types of evidence, as shown in **Table 1**. In general, evidence for lower levels is more theoretical or qualitative and that for higher levels is more empirical or quantitative, whereas in-between levels tend to have mixed evidence. For validation of individual claims with theoretical evidence, formal logic such as deductive and inductive reasoning can be adopted. For those claims with a large amount of possibly conflicting evidence (e.g., the fitness claim), one might resort to informal logic such as Toulmin’s (1958) model of inference. Note that some claims (e.g., administration) can be implicitly assumed unless challenged.

**Table 1:** Evidence for validity as defined by claims in each hierarchical level.

Level	Claim	Description	Types of evidence
Operational	Population	The population of examinee is appropriately defined	Theoretical or expert analysis
	Structure	The dimension and scale of the construct are appropriately defined	Theoretical or expert analysis
	Content	The substantive content of the construct is appropriately operationalized	Qualitative (e.g., content analysis)
Measurable	Item and format	The items and format are appropriate for the construct domain	Item analysis and qualitative (e.g., think aloud protocol)
	Specifications	The test specifications are appropriate for the construct domain	Expert analysis
	Method	The method used to analyze the data and derive the test score is appropriate	Theoretical or empirical (e.g., standard setting)
Realizable	Administration	The testing process is appropriately administered	Qualitative (e.g., independent observation)
	Fitness	The method or model fits the response data appropriately	Various fit indices or empirical analysis
Useful	Use validity	The score use (extended or joint) is appropriate	Empirical (e.g., correlation analysis)

#### Construct, Test Score, and Instrument

While the construct domain and test score are uniquely bound, the instrument and test score are separated. Test scores with the same construct domain can be equated and are considered equivalent, even if they are obtained using different instruments (e.g., different items or formats) or methods. Note that any mathematical transformation of one test score does not create a new test score (e.g., normal *z*-score vs. percentile ranking) in general, given that the structure remains. Similar concept of score transformation based on the normality assumption can be also found in Sartori (2006). Thus, while every test score connects to a unique construct domain, the connection can proceed through different means (e.g., different instruments and/or methods) and the corresponding test scores are interchangeable, given that the meaning validity holds. On the other hand, an instrument can be used to generate different test scores (e.g., norm-referenced vs. criterion-referenced, unidimensional vs. multidimensional), each of which needs to be individually validated. Alternatively, it is possible to validate one test score obtained with multiple instruments. Accordingly, the taxonomy also implies the separation of the instrument from the test score, in addition to allowing for the separation of score meaning and score use.

#### Testing Purpose vs. Foundation

Testing purpose is closely related to the intended use of the test score. In educational measurement, for instance, the purposes can be summative or formative, which can be further classified as selection, diagnosis, classification, placement, or evaluation of instructional effectiveness (Schmeiser and Welch, 2006). Different purposes can affect the use validity of the test score. Similarly, a high-stakes purpose can invalidate the use of test scores intended for a low-stakes purpose. In comparison, the testing foundation is the direct source of the operational level of the construct domain. However, purpose and foundation are connected, and it should be possible to identify a legitimate theoretical and/or practical foundation of the construct domain from a reasonable testing purpose. If the purpose is ambitious, with wide coverage of the substantive content and population, multiple construct domains, each with its own foundations, can be shaped, and a joint use of the test scores should be validated.

### Test Validation and Development

It is straightforward to streamline test validation for specific testing practices following the hierarchy of the taxonomy. The first step is to establish the operational level of validity based on the testing purpose and foundation. The second step is to establish the measurable level based on the operational definitions. The third step is to establish the realizable level based on the instrument and method. The last step is to establish the useful level, or to validate the score use for the purpose. For a joint use of test scores, each test score with its own construct domain is validated separately in the first three steps, whereas the joint use of the test scores is validated simultaneously in the last step. The operational and measurable levels are relatively stable once established, whereas the realizable and useful levels need to be established during each testing process.

In addition to its usefulness in test validation, the hierarchical taxonomy is useful for informing test development across a variety of scenarios. In general, multiple logical, though not necessarily temporal, development stages can be streamlined. In the preparation stage, testing foundation is identified, separated from testing purpose, and then used to shape the construct domain with the three elements. In the construction stage, the instrument is constructed, including its specifications, items, and response formats. The related method to analyze the response and derive the test score is also developed. Note that one can go back and forth between the preparation and construction stages, especially when the testing foundation is more practically than theoretically dominated. For classroom assessments based on practical course requirements, for instance, the construct domain can be adjusted based on the items developed for the course. In the administration stage, the test is administered based on a standardized process and the response data are analyzed for fitness. Similarly, the administration and construction stages can be iterative in practice. In the use stage, the test score is used, likely with other variables or test scores, to satisfy the testing purpose. Note that the above stage classification is not definitive. For large-scale or small testing, the stages can be further partitioned or combined. For classroom assessments, for instance, the preparation and construction stages can be combined, or the administration and use stages can be integrated with course instruction.

Conventionally, the instrument is the focus during test development. However, it is sensible to document, likely in the test manual, all important elements related to the taxonomy such as the content, population, different structures and methods to obtain different test scores, possible concerns during administration, and possible uses.

## Real-Life Examples

To demonstrate the usefulness of the taxonomy, this section provides examples of the definition and validation of construct domains for different testing practices. This information is also summarized in **Table 2**.

**Table 2:** Usefulness of the taxonomy with different real-life examples.

No	Testing purpose and foundation	Operational level (construct domain)			Measurable level		Administration concern	Score use
		Population	Structure	Content	Instrument	Method		
1	Classification and practical	Time: within year; place: regional; prerequisite	Unidimensional, criteria- referenced (certified or not)	Practice proficiency based on regulations or requirements	*Certification and licensure tests*	Criteria-driven cutoffs; multiple forms	Security; standardization	Basic (criteria-driven)
2	Academic prediction and theoretical	Years: 6–16; place: United States; time: since 2003	Unidimensional and norm-referenced	Intelligence (verbal comprehension, perceptual reasoning, working memory, processing speed)	*WISC-IV*	Composite score based on reliability;factor analysis	Balancing the wide coverage of content and test time	Extended or joint
3	Diagnosis and theoretical	Same as above	Multidimensional and categorical (mastery/partial/Non-mastery)	Various dimensions (subtests): e.g., block design, digit span, vocabulary, picture concepts, arithmetic	*WISC-IV*	Criteria-driven cutoffs	Same as above	Basic (criteria-driven)
4	Placement and practical	Time: current year; age: specific grade; place: regional	Unidimensional and categorical (levels of competencies)	Competencies based on course requirements	*Placement test*	Criteria-driven cutoffs	Usually little	Basic (criteria-driven)
5	Placement and practical	Same as above	Unidimensional and norm-referenced	Same as above	*Placement test*	CTT- or IRT-based	Same as above	Extended (selection-driven with other factors)
6	Admission and theoretical	Time: within year; place: non-native	Unidimensional and norm-referenced	English reading competencies	*TOEFL Reading*	IRT-based	Security; standardization	Joint (e.g., with listening and speaking)
7	Admission and mixed	Same as above	Unidimensional and categorical (levels of competencies)	English writing competencies	*TOEFL Writing*	Criteria- driven cutoffs; scoring consistency	Security; rater training	Same as above

*WISC-IV, Wechsler Intelligence Scale for Children–Fourth Edition; TOEFL, Test of English as a Foreign Language; CTT, classical test theory; IRT, item response theory.*

### Certification and Licensure Testing

Certification and licensure testing practices are widely used to assess whether examinees possess the knowledge or skills necessary to perform the domain behavior (e.g., professio the hierarchical taxonomy, the first step is to shape and validate the operational definitions of the construct domain based on practice regulations or requirements. As shown in Row 1 of **Table 2**, the population should be confined by time (e.g., within years of testing) and place, as established by the regulations or requirements. Educational or professional prerequisites might be relevant as well. The structure is usually unidimensional, criteria-referenced, and dichotomous (e.g., certified or not). The substantive content should be defined by practice proficiency consistent with the regulations or requirements.

The next step is to validate the instrument for certification and licensure testing, including the items, response format, and specifications, all of which should be consistent with the operational definitions. As the structure is criteria-referenced, standard-setting methods for educational measurement (Hambleton and Pitoniak, 2006) should be used to set the cutoff criteria empirically. When multiple test forms exist, determining how to equate test scores from different forms should also be a method concern. The largest administration concern relates to standardization and security, especially when the tests can be given at different places and/or times and the purpose has high stakes. The score use is basic because the certification or licensure decision is based on criteria-driven cutoffs and no other variable is involved. For the validation process, qualitative evidence can be collected, such as expert agreement on the content and items or empirical evidence for the cutoff to support or reject the associated claims.

### Intelligence Testing

As mentioned above, one instrument can be used for different purposes, and each purpose needs to be individually validated. The Wechsler Intelligence Scale for Children (WISC; Wechsler, 1949, 2004) has been widely used as a measure of intelligence. Based on the updated technical documents (Williams et al., 2003a,b), it appears that the instrument is flexible enough to help define different test scores with specific construct domains for different purposes. The two cases shown in Rows 2 and 3 of **Table 2** serve a didactic purpose. In the first case, the purpose is to predict academic success, and the test score is the full-scale score. In the construct domain, the population should be confined by age, place, and time, and the score is usually unidimensional and norm-referenced. The substantive content should include various areas such as verbal comprehension, perceptual reasoning, and working memory. Accordingly, the instrument that is consistent with the construct domain should cover a variety of item types that are appropriate for the content and target population (Williams et al., 2003a). For the method part, a composite score with *z*-score transformation can be adopted based on evidence of high reliability coefficients, and factor analytic models can be used to show homogeneity across subtests. The biggest administration concern is to balance the wide coverage of content and the time required to finish the test. For prediction purposes, the test score can be used together with other variables and/or test scores.

In the second case, the large number of subtests for various cognitive functions (Williams et al., 2003a) can be used to shape a construct domain for cognitive diagnosis or intraindividual comparisons. Accordingly, there is a need to validate a large number of dimensional definitions in the content element, each corresponding to a specific cognitive subtest (Williams et al., 2003b). Although the population element is basically unchanged, the structure is multidimensional and criterion-referenced based on criteria such as mastery, partial mastery, and non-mastery for each dimension. The instrument and administration parts can be validated similar to the way described above, but the method and score use parts should be different with methods appropriate for a criterion-driven cutoff and a basic score use.

### Placement Testing

Placement testing is widely used to place students into different levels (e.g., regular vs. advanced) of related courses according to the students’ competencies (van der Linden, 1998). Under the hierarchical taxonomy, two construct domains can be defined and validated based on the types of decisions: criteria driven or selection driven (Rows 4 and 5 in **Table 2**). The major differences stem from the structure element. In the criteria-driven case, the levels of competencies correspond to the levels of the courses, and students are placed into the corresponding courses once they meet the criteria, regardless of the number or proportions of students occupying the different levels. This implies that resources, such as number of classes or class sizes, are flexible enough to accommodate fluctuations of competent students across levels. In contrast, placement decisions are based on both the test score and other factors (e.g., class sizes) in the selection-driven case, for which it would be better to adopt a norm-referenced scale, like percentile ranking. The limitation of resources becomes part of the evidence for use validity, and factors such as fairness or equality may need to be considered to justify the score use.

### Large-Scale Testing

In large-scale testing, it is likely that the intended content differs substantially across areas, which can pose challenges for validation using one construct domain or test score. Here, the Test of English as a Foreign Language (TOEFL; Educational Testing and Service, 2010) illustrates how multiple test scores can be defined and evaluated based on the taxonomy. Instead of validating one construct domain summarizing four skills (i.e., reading, writing, listening, and speaking), it may be better to define each skill as an individual construct domain for two reasons: (a) the testing foundations related to specific skills differ substantially, and some skills (e.g., writing) can be less theoretically founded than others (e.g., reading); and (b) the scores for different skills can use different frames of reference (i.e., norm-referenced vs. criterion-referenced). Using reading and writing skills as examples, it is possible to define two construct domains with different structure and content elements, as shown in **Table 2**. Accordingly, while the reading test score is norm-referenced based on objective items, the writing score is criterion-referenced based on open-constructed items. Once the score meaning for each skill is validated, the four test scores, each with its own construct domain, can be used together, likely with variables (e.g., high school grade-point average) for decision making related to college admissions. In this case, it is mandatory to provide validity evidence for the score meaning (e.g., how the criterion-driven cutoff is empirically established or the scoring between raters is consistent) and joint score use (e.g., for variable choice and the specific compensatory or conjunctive way the test scores and variables are combined).

## Discussion

This paper has proposed a taxonomy to redefine test validity with hierarchical levels. On the basis of testing foundation, the hierarchy includes the operational, measurable, realizable, and useful levels, which result in testing consequence. With the help of a context-specific construct, different levels of test validity, and three types of score use (i.e., basic, extended, and joint), the proposed taxonomy offers great flexibility for test validation and aids in the understanding of test validity from a different perspective. Among others, it is featured with a hierarchy of validity, unique score meaning, the separation of meaning and use validity, and joint validation of heterogeneous contents with multiple test scores. Note that, more often than not, a test score is not used alone. Accordingly, a mix of extended and joint use can provide even more flexibility. With these, a variety of educational and psychological testing practices ranging from classroom assessments to large-scale settings can be effectively accommodated. The taxonomy can also shed light on the reinterpretations of important testing concepts and different logical stages in test development. During test development, it is sensible to organize various information and evidence around the taxonomy systematically, likely in the test manual or assessment framework.

Validity lies at the core of the complicated educational and psychological testing enterprise. Accordingly, in applications of the proposed taxonomy, there is need for careful and consistent application of this logic in test evaluation, test development, and measurement across various settings. Test validity should be regarded as one part of test evaluation, which also includes test reliability and the evaluation of testing consequences. It is generally agreed that test reliability is a basis of test validity. What test reliability actually refers to is the reliability of score meaning. Namely, test reliability identifies the extent to which the score meaning is reliable within the same context of the construct domain, which is prerequisite to the validity of score meaning. On the other hand, test validity serves as a basis of consequence evaluation. It is meaningless to talk about testing consequences before validating the score meaning and use. Meanwhile, the positive or negative consequences of score use are largely value laden, whereas test validity is essentially scientific.

The roles of test developer and user should be separated in test evaluation and development. In general, the test developer is responsible for the validity of score meaning and the intended score use. In contrast, the test user should validate and justify the score use, especially when it is different from the use intended by the developer. Note the difference between the use of test score only (e.g., secondary analysis) and the use of the instrument and test score. In the latter case, the user should also provide evidence to support the administration and fitness claims. Measurement is intertwined with test validity, but the degree of involvement differs across the hierarchical levels of the taxonomy. In general, the involvement is deeper when more quantitative or empirical evidence is required, and is lighter if qualitative or theoretical evidence dominates.

The major challenge to implement the taxonomy might be related to the difficulty to define and validate the operational level of the context-specific construct, especially when the theoretical foundation is weak or the practical one is not solid enough. The reason comes from the understanding that test validity is conditional on the legitimacy of the testing foundation under the taxonomy. In reflection, this is consistent with the methodological nature of test validation under the epistemological approach and cross-disciplinary efforts are always indispensable. As regarding future directions, more details about each level of the taxonomy can be further substantiated, likely together with specific guidance of implementation across various settings. It would be meaningful to investigate the similarity and difference between the proposed taxonomy and other validation approaches through empirical studies. It might be also useful to structure the various measurement processes and frameworks in relation to the taxonomy in future research.

Among the contextual factors that can be used to define a context-specific construct, time is the one that required special attention since many behavior patterns change substantially over time. Incorporation of the time factor implies that the score meaning can be redefined periodically depending on how behavior patterns evolve (Chen, 2017). However, this way of redefining score meaning is not gradual but, rather, episodic in a way similar to the paradigm shift of scientific progress (Kuhn, 1962). In this case, redefining and validating the new score meaning is somewhat similar to a micro version of scientific progress.

Based on the definition of psychology as the study of behavior and constructs or attributes (Crocker and Algina, 1986), the epistemological approach of test validity can be connected to the contemporary situational perspectives of educational psychology, which also favor the interdependency and interaction of individual behavior and contextual factors (Greeno et al., 1996; Mason, 2007). Similarly, the ontological approach can be associated with the traditional cognitive perspective, where individual behavior is explained as a function of the underlying attribute. In this regard, this research provides methodological feasibility in response to Mason’s (2007) charge of bridging the two perspectives of educational psychology. The utility of the taxonomy, however, can be examined only with empirical applications. Considering the methodological nature of the taxonomy, successful applications would be elusive without collaborative effort across disciplines.

## Author Contributions

The author confirms being the sole contributor of this work and approved it for publication.

## Conflict of Interest Statement

The author declares that the research was conducted in the absence of any commercial or financial relationships that could be construed as a potential conflict of interest.

## Footnotes

1 Note that while the terms *construct* and *attribute* are often used under the epistemological and ontological approaches, respectively, they are treated as interchangeable in this article.

2 One test score includes one set of scores in multidimensional forms (e.g., multiple subscale scores) or in vector form (e.g., attribute profile).
